# Recent Advances on Antimicrobial and Anti-Inflammatory Cotton Fabrics Containing Nanostructures

**DOI:** 10.3390/molecules26103008

**Published:** 2021-05-18

**Authors:** Albert Granados, Roser Pleixats, Adelina Vallribera

**Affiliations:** Departament de Química and Centro de Innovación en Química Avanzada (ORFEO-CINQA), Facultat de Ciències, Universitat Autònoma de Barcelona, UAB Campus, C/ dels Til.lers, 08193 Cerdanyola del Vallès, Spain

**Keywords:** anti-inflammatory, antimicrobial, cotton fabrics, functionalization, nanoparticles, textiles

## Abstract

Hydrophilic cotton textiles, used in hospitals and sportswear, are prone to the growth of microorganisms (bacteria, fungi) resulting in hygiene and health risks. Thus, healthcare concerns have motivated the interest for the development of multifunctional antimicrobial cotton fabrics. Moreover, cotton textiles are also used in medical applications such as wound dressings. Their functionalization with anti-inflammatory agents is desirable in order to accelerate cicatrisation in the treatment of chronic wounds. This review summarizes recent advances (from January 2016 to January 2021) on the modification and coating of cotton fabrics with nanostructures (mainly metal and metal oxide nanoparticles, functionalized silica nanoparticles) to provide them antimicrobial (antibacterial and antifungal) and anti-inflammatory properties.

## 1. Introduction

Cotton, a low-cost and natural fiber, which is formed of practically pure cellulose, is of great importance in the apparel industry due to its unique combination of properties, such as softness, strength, elasticity, biodegradability, water affinity and permeability. However, hydrophilic cotton fabrics are more prone to microbial degradation than synthetic fibers as they offer more favorable conditions for the growth of microorganisms such as bacteria and fungi, resulting in deterioration of textile strength, unpleasant odors, allergic responses and hygiene and health risks. This contamination occurs mostly in textiles used in hospitals, sportswear and underwear, which often may have contact with bacteria and fungi. Thus, healthcare concerns have motivated a growing interest of researchers for the development of multifunctional textiles that can inhibit the growth of microbes [1].

Surface modification of cotton fibers with biopolymers (chitosan, starch) and other synthetic polymers to impart antimicrobial activity and overcome other limitations of this natural textile has been reported [2]. Quaternary ammonium salts, industrial enzymes and several metal salts have also been described as agents for the development of antimicrobial textiles [3] as well as the use of other antimicrobial products (polyhexamethylene biguanide, triclosan, *N*-halamine, peroxyacids, dyes) [4,5,6]. Pyrazole-based compounds have been encapsulated into liposomal chitosan emulsions for textile finishing and the treated cotton fabrics exhibited activity against gram positive and negative bacteria [7]. 

The nanotechnology has been incorporated in the last decades to the production of antimicrobial fabrics. The influence of nanofinishes (zinc oxide, titanium oxide and silver oxide nanoparticles, silver nanocolloids) on the antimicrobial properties of natural (cotton, wool, silk) and some synthetic fibers has been remarked [8]. The antibacterial effects of different metal and metal oxide nanostructures on a wide range of natural and synthetic fibers has been reported [9]. Specially, zinc oxide nanoparticles [10] and copper(II) oxide nanoparticles [11] have been extensively studied for the obtention of antimicrobial functionalized textiles. Furthermore, silver nanoparticles are one of the most explored nanomaterials for applications in biomedicine due to their biocompatibility and nontoxicity; they have been the subject of a recent review [12] in which their antibacterial, antiviral and antitumor activity have been highlighted together with their use in tissue engineering and wound care. Coating of textiles with chemically or physically modified silica nanosols have also been reported to provide antimicrobial activity among other properties such as hydrophobicity, oleophobicity, anti-adhesive, UV-protection [13]. Solution chemistry methods allow the combination of the synthesis of metal oxide nanoparticles (TiO_2_) with natural or synthetic textile finishing processes [14]. 

The antimicrobial durability of cotton textiles modified with inorganic nanoparticles (Ag, Ag_2_O, AgCl, ZnO, TiO_2_, ZrO_2_, Cu_2_O, CuO and MgO) has been investigated. The use of suitable polymer binders and microwave or ultrasound techniques have been found effective ways to improve the durability, preventing the loss of the nanoparticles during the washing process [15]. 

Antimicrobial activity has been related to superhydrophobic nanomaterial coating due to synergistic effects. Thus, metal and metal oxide nanoparticles cause damages in microbes, also preventing the adhesion of the microorganisms on superhydrophobic surfaces. Hence, the surface morphology must be optimized in such a way that the superhydrophobicity and the antimicrobial ability are maximized [16]. 

Moreover, cotton fabrics are commonly used for medical applications such as wound dressing. In this sense, apart from the need to avoid microbial degradation of the medical textiles, functionalization of bandages, gauzes and wound dressings with anti-inflammatory agents is desirable in order to accelerate cicatrisation in the treatment of chronic wounds. Even though much less explored, this subject is promising for topical cutaneous applications in dressings [17]. Recently, lipid systems containing omega-3 fatty acids (nanostructured lipid carriers, NLCs) or omega-3 fatty acid and resveratrol (liposomes) have been incorporated into woven, non-woven and knitted cotton substrates by exhaustion and impregnation. From the evaluation of the release profiles, the textiles are expected to be useful for wound healing and anti-inflammatory applications [18].

This review article focuses on recent advances (from January 2016 to January 2021) dealing with the modification and finishing of cotton fabrics with nanostructures (mainly metal and metal oxide nanoparticles, functionalized silica nanoparticles) to provide them antimicrobial (antibacterial, antifungal) or anti-inflammatory properties. 

## 2. Discussion

### 2.1. Antibacterial Cotton Fabrics Containing Nanostructures

Bacterial infections and their related complications can even lead to the death of patients. With the aim to outstrip the limitations that take place due to drug-resistant pathogens, worldwide researchers focused on the development of unconventional antibacterial strategies [9,19]. Amongst them, nanomaterial-based formulations represent a feasible choice for modern antibacterial agents.

#### 2.1.1. Metal Nanoparticles (M NPs)

Silver nanoparticles are one of the most promising metal-based bactericidal that have been used in medicine [12]. Silver endows high antibactericidal action even with small dose usage and it is not harmful to human being. Most scientists have attributed inhibition of bacterial cell due to the presence of Ag^+^ ions upon dissolution of Ag NPs inside or outside the bacterium [12].

There are several application techniques that have recently been used for coating Ag NPs on cotton fabrics. However, it has been recognized that the deposition on textiles is not permanent particularly due to washing processes. Application of binders, cross-linkable polymers and agents have been adopted. 

The synthesis of Ag graphed textile via pad coating impregnation method (pad-dry-cure) has been used by many researches via wet chemical or biomass filtrate. Ag NPs are first prepared before padding the textile into NPs colloidal solution, drying and finally curing. The green syntheses of nanoparticles using actinomycetes, fungi, bacteria, algae and plants have better advantages as they are environmentally safe. In 2016, it was reported that cotton fabrics could be treated with a solution containing *Tragacanth gum*, as a natural polysaccharide polymer, silver nitrate, citric acid (cross-linker) and sodium hypophosphite (catalyst) to prepare a *Tragacanth gum*/nanosilver hydrogel on cotton fabric [20]. Antibacterial activities were observed, reducing the number of colonies against *E. coli* and *S. aureus* (Table 1, run 1). 

**Scheme 1:**
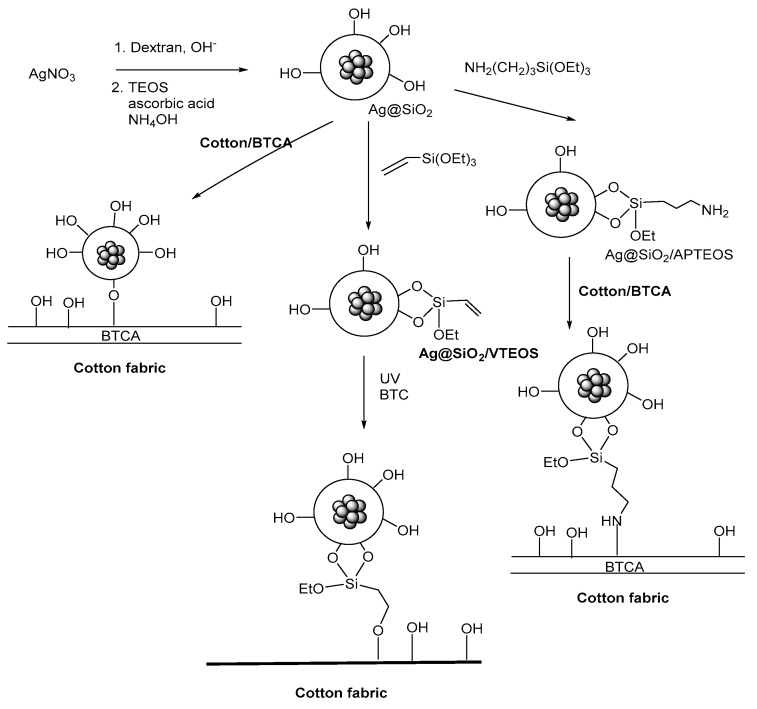
Preparation of Ag NPs functionalized with different silanes and covalent reaction onto cotton surface.

Searching for advanced functionalization of cotton textiles, Ag NPs were prepared from silver nitrate using the biopolymer dextran as reducing and stabilizing agent and then added to a tetraethoxysilane (TEOS) solution that, after aging, formed a solid Ag@SiO_2_. These Ag@SiO_2_ were modified with other silane compounds (vinyltriethoxysilane (VTEOS) or (3-aminopropyl)triethoxysilane (APTEOS). Ag@SiO_2_ and Ag@SiO_2_/APTEOS were applied to cotton fabrics using 1,2,3,4-butantetracarboxylic acid (BTCA) as a cross-linking agent and a laboratory paddler as the technique of coating. In addition, the functionalized fabric with Ag@SiO_2_/VTEOS was treated, using UV irradiation, with 4-benzoyl(benzyl)trimethylammonium chloride (BTC) as initiation for polymerization. The in vitro antibacterial activity of treated cotton fabrics was quantitatively determined against two types of bacteria *E. coli* and *S. aureus*. Treated cotton fabrics with Ag NPs have an excellent antibacterial activity. However, the bacterial reduction was decreased with increasing the washing cycle. It has been found that the antibacterial activities of the fabrics treated with Ag@SiO_2_/VTEOS and with Ag@SiO_2_/APTEOS have the same values for both *E. coli* and *S. aureus,* however Ag@SiO_2_/VTEOS is the most resistant to washing cycles (Scheme 1, Table 1, run 2) [21]. 

**Scheme 2:**
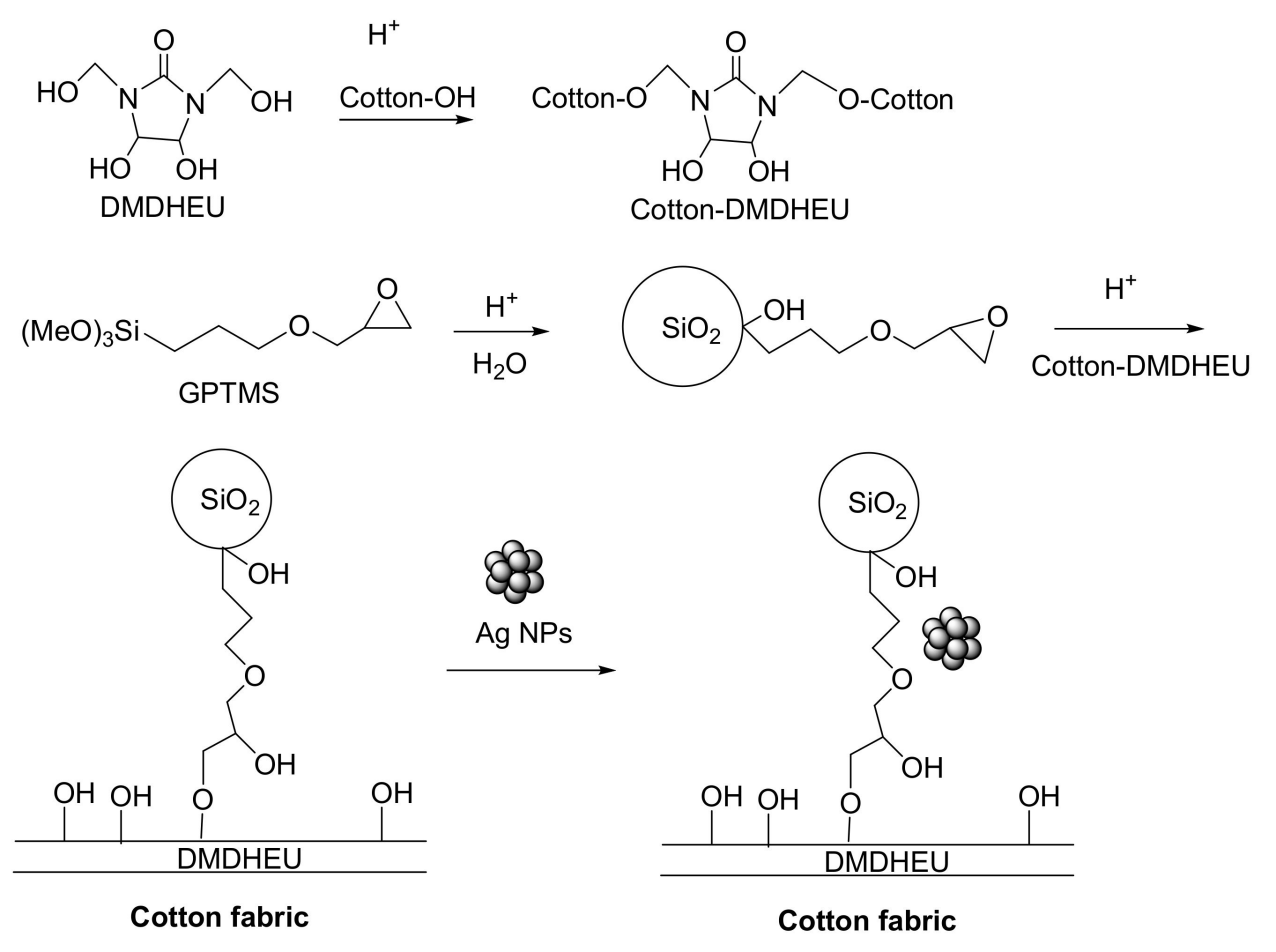
Preparation of functionalized cotton fabrics using DMDHEU and GPTMS as cross-linkers and fixing agents.

Ibrahim and collaborators have published the treatment of cotton fabrics with a prepared sol by a padded process. Dimethylol dihydroxyethylene (DMDHEU) was added as a cross-linker that reacted with the cotton hydroxylic groups forming cotton-DMDHEU. Simultaneously, a silica sol form was prepared from 3-glicidyloxytrimethoxysilane (GPTMS), which was covalently linked to cotton-DMDHEU by reaction of hydroxylic groups with the epoxides in acid media. Ag NPs were prepared from silver nitrate and carboxymethyl cellulose as reducing and stabilizing agent (Scheme 2). Fabrics were padded twice. Sometimes a fluorinated finishing water repellent agent was added. Then, Ag NPs were added [22]. Antibacterial activity was successfully evaluated against S. *aureus* and *E. coli* (Table 1, run 3).

Another process of deposition consisted in immersion of textiles in silver containing solutions. As an example, the group of Seino in 2016, irradiated with a high-energy electron beam an AgNO_3_-soaked textile. This process induces a reducing reaction forming Ag NPs [23] due to the species generated by radiolysis of water and by the irradiated fabric. The Ag NPs immobilized on the support textiles fabrics exhibited an excellent antibacterial activity across a wide antibacterial spectrum (*S. aureus*, *Klebsiella pneumoniae*, *MRSA*, *E. coli*, *P. aeruginosa*, *Salmonella enterica* and *Vibrio parahaemolyticus)* (Table 1, run 4).

Recently, there has been an increasing interest in implementing plasma technology, as an eco-friendly physical tool, to modify and activate the surface properties creating new active sites. Conductive polymers can produce conductive surfaces by plasma-assisted coating in the presence of silver nanoparticles introducing antibacterial activity and electrical conductivity. A pre-surface modification of the cotton fabric using N_2_-plama to create new active binding sites (-NH_2_ groups) followed by subsequent loading of biosynthesized Ag NPs has been described by Ibrahim [24]. The cellulosic substrate was pre-activated through placing it between the two electrodes of dielectric barrier discharge plasma, using N_2_ gas. Then, the N_2_-plasma treated fabric was post-treated with the Ag NPs dispersion, biosynthesized using AgNO_3_ as precursor and marine bacterial as reductant (*Bacillus *sp.). Subsequent loading with certain antibiotics was also assayed, such as Ciprofloxacin^®^ and Cefobib^®^. This post-treatment step was accompanied by a remarkable improvement in the imparted antibacterial activity against *S. aureus* and *E. coli* pathogens regardless of the used antibiotic type, which reflects the synergy between Ag NPs and the antibiotic (Table 1, run 5). Moreover, the imparted antibacterial activity against *S. aureus* and *E. coli* pathogens was still retained even after 15 washings. Another recent example based on plasma activation of the surface was described by Ahmed and Rehan [25]. The plasma treated cotton fabric samples (glow discharge plasma under atmospheric pressure) were activated and then soaked in a solution of aromatic amine, ammonium acetate and silver nitrate. They report polymerization of the aniline to afford thin films of conductive polymers. Silver nitrate was simultaneously reduced by the aromatic amine to Ag NPs. As expected, polyaniline conductive polymer/AgNPs coated onto plasma cotton fabrics demonstrated improved antibacterial activity compared to polyaniline conductive polymer/cotton samples against *E. coli* and *S. aureus* (Table 1, run 6). 

**Scheme 3:**
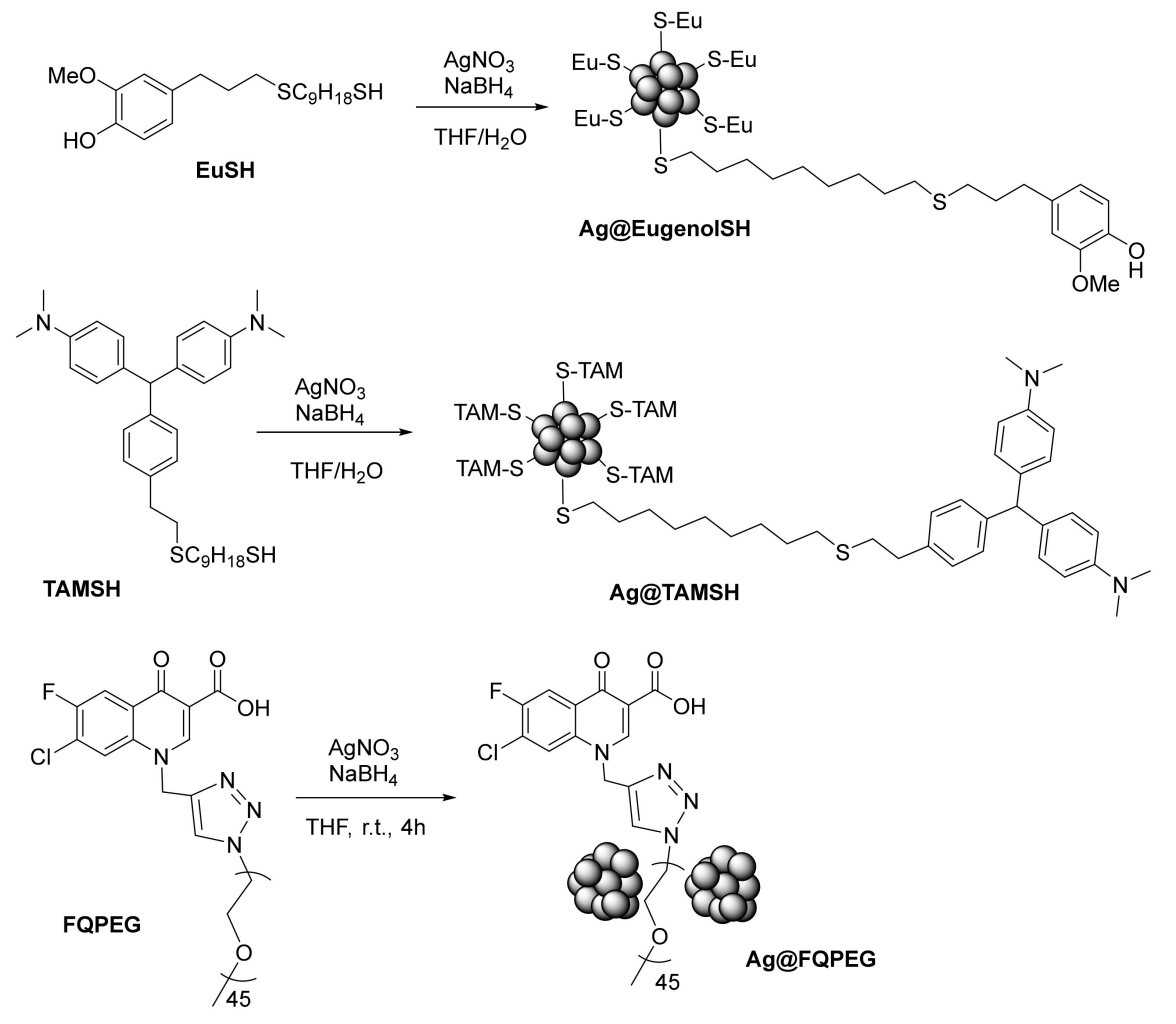
Preparation of Ag NPs using different antibiotics as stabilizing agents.

We have used functionalized antibiotics derived from Eugenol (Eu), diaminotriarylmethane (TAM) and fluoroquinolone (FQ) as stabilizers to protect Ag NPs (Ag@Antibio). These nanoparticles were prepared mixing a solution of AgNO_3_ and NaBH_4_ as reducing agent in the presence of the different selected antibiotics derivatives as stabilizers. Microbicidal activity studies of fabricated cotton textiles coated by impregnation with these Ag@Antibio were performed. Protective ligand layers of Ag NPs resulted to be a deterministic factor in their properties. The best bactericidal activity was obtained for fabric coated with Ag NPs with diaminotriarylmethane derivates (Ag@TAMSH) in surface against *S. aureus* strain. Intrinsic antibiotic activity and partial positive charge of the ammonium salts of the TAMSH in the surface of the NPs probably enhanced their antimicrobial effects. Fabric coated with Ag NPs with eugenol derivates in surface (Ag@EugenolSH), and fabric coated with Ag NPs embedded in PEG-fluoroquinolone derivatives in surface (Ag@FQPEG), displayed antibacterial activity for both *S. aureus* and *P. aeruginosa* strains [26] (Scheme 3, Table 1, run 7).

**Scheme 4:**
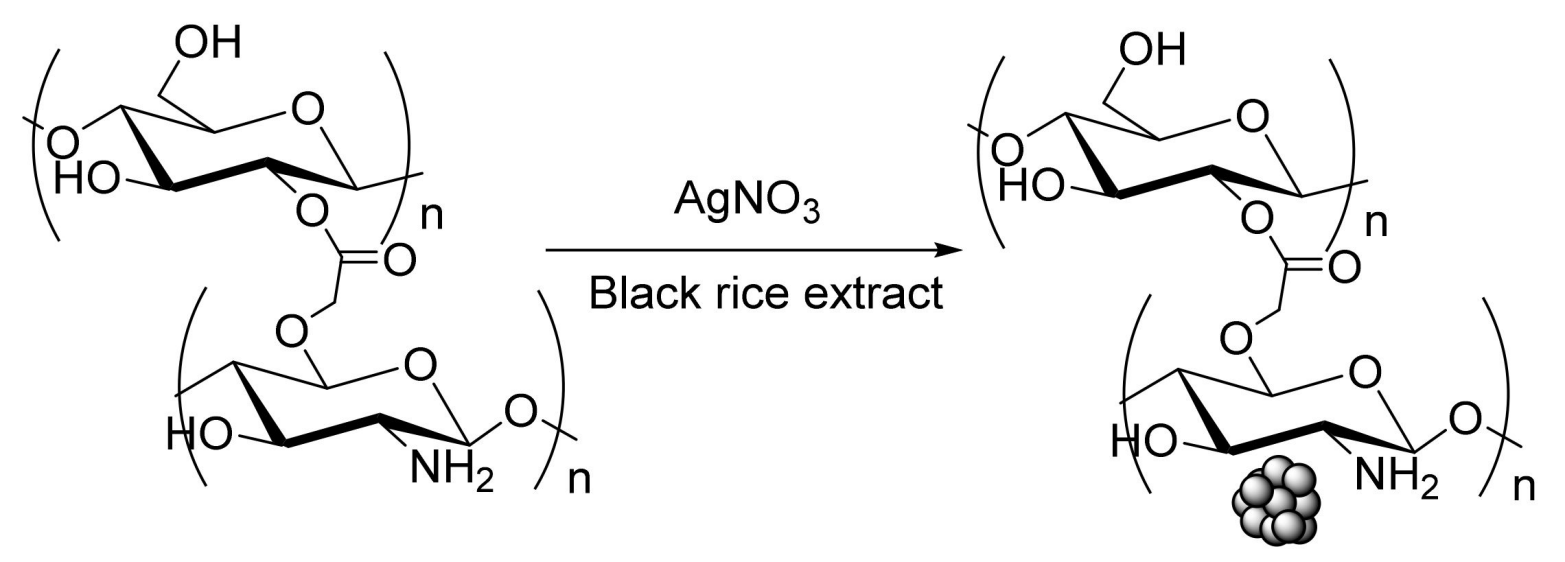
Ag NPs onto cotton fabric modified with carboxymethylchitosan surface.

Another interesting example of impregnation of Ag NPs onto a fabric surface by dipping is due to He’s group [27]. The cotton fabric was first padded with a solution of carboxymethylchitosan and then soaked in a solution of silver nitrate and black rice extracts (reductant). The Ag(I) is coordinated with the donating groups of chitosan (-OH and -NH_2_) and anthocyanines metabolites reduce this Ag(I) to Ag(0). This novel method imparted cotton fabric with excellent antibacterial ability against *E. coli* and *S. aureus* (Scheme 4, Table 1, run 8).

Sonochemical preparation provides a facile approach to produce spherical metal nanoparticles. Ultrasonic irradiation of water generates highly reactive H and OH radicals, which are responsible for redox chemistry. Baytekin’s group prepared cotton Ag NPs composites via ultrasonication. Fabric-metal composites were prepared dipping cellulose samples on AgNO_3_ solutions in water. The formation of radicals from water during the sonication of cellulose has been determined by ESR studies. Ag NPs loaded on the fabrics inhibited the growth of *E. coli* and *B. subtilis*, yet they were more potent against *E. coli* in comparison to *B. subtilis* [28] (Table 1, run 9).

Aerosols of silver nanoparticles were produced by means of electrical discharges between two electrodes in nitrogen and passed through fabrics were particles are retained. High purity silver electrodes were used. The antibacterial activity of the fabrics coated with silver nanoparticles were studied with success against *S. aureus* and *Klebsiella pneumoniae* [29] (Table 1, run 10).

In addition to silver, there are other metals of interest that have been studied. Among them, copper. Aerosols of copper were produced by means of electrical discharge in nitrogen and passed through fabrics were particles are retained, in the same conditions that Ag NPs. The antibacterial activity of cotton samples with copper NPs was assessed against *S. aureus* and *Klebsiella pneumoniae* in compliance, obtaining only acceptable antibacterial results [29].

In addition, gold has attracted special interest due to the Zheng et al. [30] suggestion that Au NPs coated on fabric possess UV protection and antibacterial properties. A greener synthesis of gold nanoparticles was successfully done by reducing HAuCl_4_.3H_2_O using *Coleus aromaticus* leaf extract. These stabilized Au NPs were coated on pre-treated cotton fabrics by simple pad-dry-cure method. The Au NPs coated fabrics exhibited remarkable antibacterial sensivity against *S. aureus* and *E. coli*. Further they showed cytotoxicity against human liver cancer (HEpG2) cell line [31]. More recently, the same group of research have synthesized phyto-engineered Au NPs from the aqueous extract of *Acalypha indica*. An equal quantity of *Acalypha indica* leaf extract and of HAuCl_4_.3H_2_O buffer solution (pH 7) were taken in an Erlenmeyer flask. The intact extract was appropriately coated to the cotton fabric employing a pad-dry-cure procedure. The gold nanoparticle-coated cotton fabric was evaluated for the antibacterial activity against *S. epidermidis* and *E. coli* bacterial strains, which revealed remarkable inhibition [32].

#### 2.1.2. Mixtures of Metal Nanoparticles

To enhance the properties of individual M NPs the generation and study of binary and tertiary nanoparticles has been developed. In 2018, bimetallic nanoparticles were generated in cotton fabrics using Aloe vera extract as reducing agent. Aqueous solutions of mixtures of AgNO_3_ and CuSO_4_.5H_2_O were first prepared and then the cotton fabrics with infused Aloe vera leaf extracts (matrix) were kept in these metallic source solutions and stirred. The nanocomposite cotton fabrics show good antibacterial activity for *E. coli*, *Pseudomonas*, *Bacillus*, *Klebsiella* and *Staphylococcus* cultures [33]. In addition, Mahesh [34] described the reduction of CuSO_4_.5H_2_O and AgNO_3_ with red sand extracts. The flavonoids and sugars of these extracts act as reducing agents and other constituents as capping agents. The cotton fabrics were dipped in the solution of the red sanders extracts and these matrices were then used to generate nanoparticles. Silver and copper nanoparticles and silver-copper bimetallic nanoparticles were prepared for comparison of antibacterial activity. Bimetallic nanoparticles generated in cotton fabrics exhibited higher activity against Gram negative (*E. coli* and *P. aeruginosa*) and Gram-positive (*S. aureus* and *B. lichinomonas*) strains.

**Figure 1:**
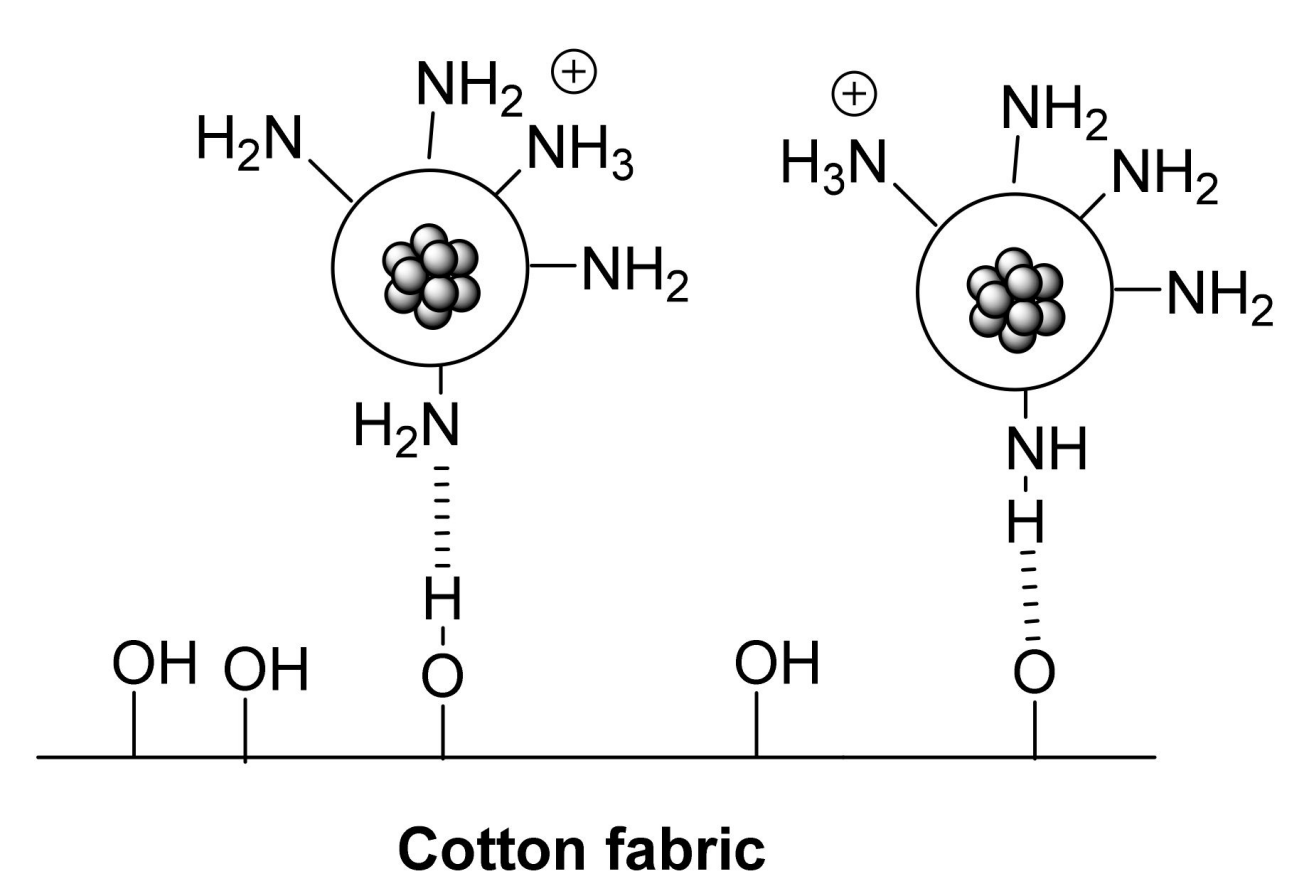
Possible mechanism of self-assembly of homogeneous hyperbranched poly(amide-amine) (HBPAA)-capped NPs of Ag, Au and Pt on cotton fabrics.

Recently, hyperbranched poly(amide-amine) (HBPAA) was used to encapsulate M NPs showing a good protective ability to prevent agglomeration and oxidation. AgNO_3_, HAuCl_4_ and H_2_PtCl_6_ were reduced by NaBH_4_ to metal NPs in the presence of the mentioned polymer. Then fibers of cotton were impregnated to produce monolayered Ag-Au-Pt coated fabrics probably due to electrostatic repulsion among positively charged NPs. Furthermore, the combination of Ag, Au, Pt nanoparticles (Figure 1) yields a positive potential and could aid in the self-assembly to cotton upon surface capping with HBPAA. Moreover, the authors [35] infer that different metal NPs capped with the same functional polymer may inherit the chemical characteristics of the latter. High antibacterial activities were found against *E. coli* and *P. aeruginosa*.

#### 2.1.3. Metal Oxide Nanoparticles (MO NPs)

The antibacterial activity of metal oxides is believed to be a consequence of their dissolution into metal ions, which can be cytotoxic to bacteria. Furthermore, as we have seen for M NPs the growth of metal oxide nanoparticles in an eco-friendly manner by plant materials has attracted significant attention. 

In 2017, ZnO NPs were prepared from Zn(AcO)_2_.2H_2_O using aqueous leaf extract of *Cardiospermum halicacabum*. The antimicrobial properties of ZnO nanoparticles coated on cotton fabrics against clinical isolates *E. coli* and *S. aureus* were evaluated [36]. Losego’s group, in 2019, examined the atomic layer deposition (ALD) of ZnO films onto cotton fabrics to control bacteria spread. ALD is a well-known technique for thin-film deposition in which vapor phase-precursors are sequentially delivered to a substrate and undergo self-limiting surface reactions, depositing a film ‘‘atomic-layer-by-atomic-layer’’. Curiously enough, at low ZnO loading, bacteria could metabolize Zn^2+^ ions and reproduced more rapidly. However, they found that increasing the thickness of the ZnO film, the nanocoating became an effective biocide material with a complete eradication of all *E. coli* bacteria present [37]. 

Moreover, aiming the preparation of bleached cotton khadi fabrics (handloom woven from handspun yarns) with UV protection and antimicrobial properties, different amounts of ZnO NPs were dispersed in poly-hydroxy-amino methyl silicone (PHAMS) and applied to these fabrics using the pad-dry-cure method [38]. This method will give rise to this modified cotton by a single-step treatment. First, the ZnO NPs were prepared by a co-precipitation method and then dispersed in poly-hydroxy-amino methyl silicone. By varying dosages of ZnO NPs (1–5%) dispersed in the PHAMS binder media (2–10%), the authors found that with 1% of ZnO NPs and 4% of PHAMS the modified materials led to an UV protection factor of 10 and 93–95% of microbial reduction (against *S. aureus* and *K. pneumoniae*). This metal combination did not provide antifungal properties. Better UV protection (factor of 20) and microbial reduction (99%) were obtained keeping the amount of PHAMS and increasing the amount of ZnO NPs up to 5%. The microbicide activity is reduced upon 96% and UV protection factor is reduced at 15 after five cycle washes. Finally, this modified bleached cotton khadi fabric is in consonance with the commercially available UV absorber 2-hydroxy-benzotriazole in terms of UV protection.

Another approach, reported by Belay, to prepare zinc oxide nanoparticles used ZnCl_2_ and NaOH aqueous solution. The particles were peptized with 2-propanol at room temperature to disrupt microagglomerates. Finally, the prepared particles were treated thermally in the oven for 5 h at 250 °C, which leads to the formation of ZnO NPs. For the application of NPs on cotton fabrics, the cotton sample was immersed into the dispersion of ZnO NPs. Another preparation reported in the same article consisted on the in situ deposition immersing the fabric in a solution of Zn(NO_3_)_2_.6H_2_O. Afterwards, NH_4_Cl, urea and ammonia solution were added to the reaction vessel. The system was heated to 90 °C and kept for 60 m. After the reaction was completed, the fabric was rinsed several times using distilled water and finally, it was kept in the oven at 150 °C to ensure particles’ adhesion to the fibers’ surface. The ZnO NPs synthesized by the two methods possess very good bacteriostatic activity against *S. aureus* and *E. coli* bacteria [39].

Recently, copper oxide nanoparticles have gained significant importance due to their distinctive properties (applications in batteries, catalysis, gas sensors and in electrical, optical and solar energy exchange tools), including that they are cheaper than other metal oxides. To reduce the risk of toxicity, *Ruellia tuberosa* aqueous extract was used for the synthesis of CuO NPs in a study reported in 2019 [40]. The fabrics were then treated with green synthesized CuO NPs and were allowed to dry at 50 °C for 30 min. Cotton fabrics showed bactericidal activity against clinical pathogens such as *S. aureus*, *E. coli* and *Klebsiella pneumoniae*. 

In a 2016 paper, Gedanken described the simultaneous deposition on cotton fabrics of Reactive Orange 16 (RO16) or Reactive Black 5 (RB5) with antibacterial CuO or ZnO nanoparticles from an aqueous solution. The solution contained both the dye and the corresponding M(OAc)_2_ (M = Zn or Cu) precursor, which undergoes hydrolysis under alkaline conditions (ammonia) to form ZnO or CuO. The cotton was colored with the dye and showed good antibacterial properties [41].

In addition, Manteccas’s group reported the coating of ZnO or CuO NPs on fabric surfaces using the roll to roll sonochemical installation. They observed solvent as one of the factors that influenced the shape and size of the sonochemically produced metal oxide NPs. The synthesis of NPs and the coating of textiles were carried out with water, which is the safer solvent in industry, as well as with a mixture of ethanol/water. Ethanol/water mixture gave less NPs leaching. Copper/zinc acetates solutions were first heated by the ultrasonic transducers, and after a temperature of 60 °C was reached, an aqueous solution of ammonium hydroxide was injected into the reaction cell to adjust the pH to ∼8.0. At the end of the reaction, a change of the color of the fabric from white to brown was observed in the case of CuO NPs, whereas, for ZnO NPs, it remained white (ZnO NPs are a white solid). Antibacterial tests revealed that, in the case of CuO NPs coated bandages in both water and ethanol, the complete killing of both *S. aureus* and *E. coli* bacteria was observed, ZnO NPs-coated bandages in water or ethanol were less effective [42].

**Figure 2:**
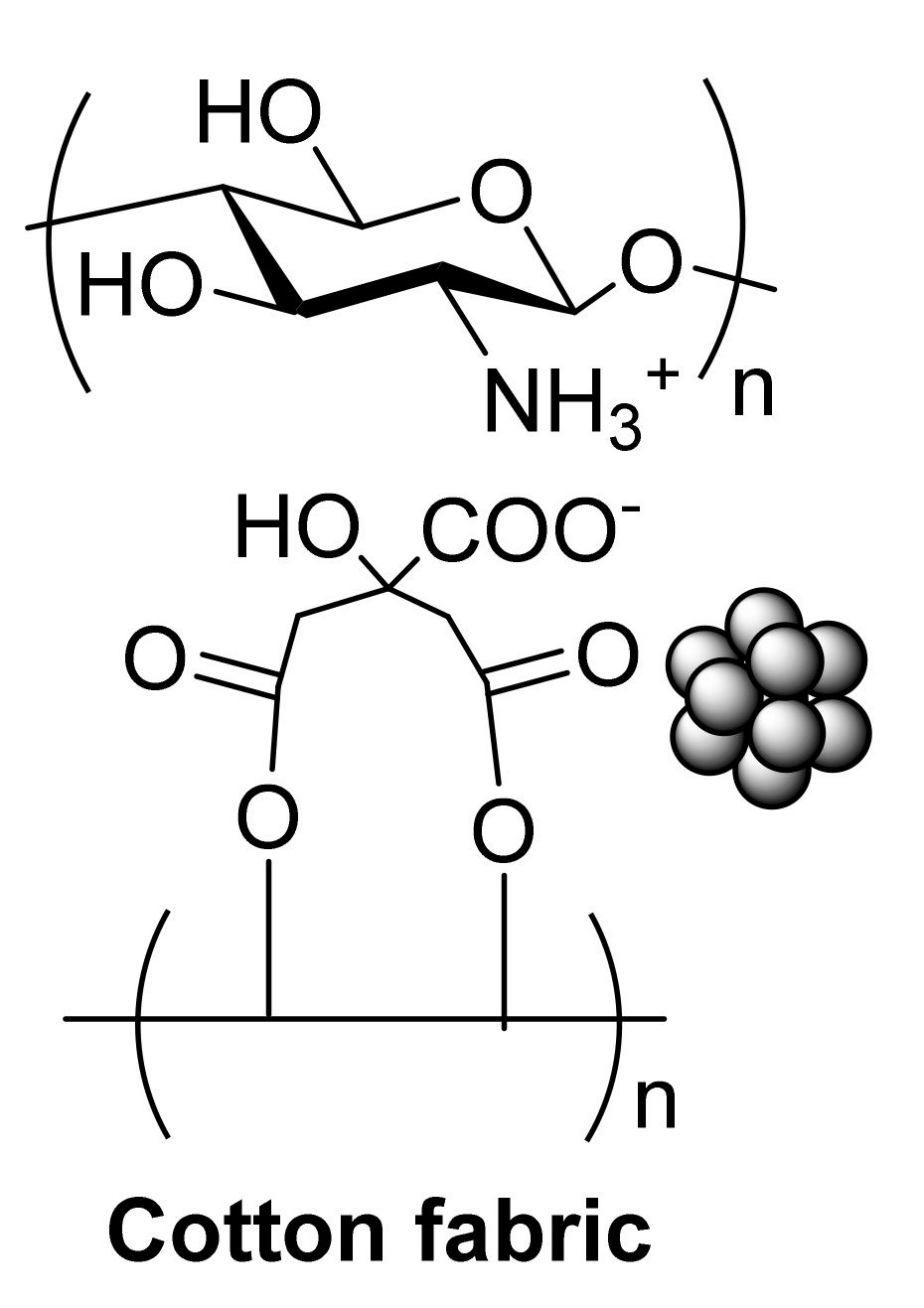
Schematic representation of anchoring sites for metal oxide nanoparticles fixation.

With the goal of preparing multifunctional fabrics Ibrahim et al. searched appropriate finishing formulations. The fabrics were paddled with 1.3-dimethylol-4.5-dihydroxyethyleneurea (DMDHEU) as crosslinking agent, MgCl_2_/citric acid as catalyst, silicone softener, UV-absorber, flame retardant and a commercial antibacterial cationic agent based on a silver compound (HEIQ^®^, Huntsman, city, Maple Shade, NJ, USA). Then embedding of the MO NPs (nano-metal oxides were of commercial grades) gave the next descending order results regarding antibacterial function: ZnO–NPs > TiO_2_–NPs > ZrO–NPs > control >> untreated [43]. Other finishing dispersions were prepared by the same group of research. In this case Chitosan (Cs) and various metal oxide nanoparticles namely ZnO, TiO_2_ and SiO_2_ were paddled onto fabric surface using citric acid/sodium hypophosphite for ester–crosslinking and creating new anchoring and binding sites, COOH groups, onto the ester-crosslinked fabrics surface. On the other hand, the extent of improvement in the aforementioned functional properties is governed by the type of nano metal oxide and follows the descending order: Cs/TiO_2_ NPs > Cs-ZnO NPs > Cs/SiO_2_ NPs > Cs alone. As a conclusion, among the used Cs/MO’sNPs hybrids, Cs/TiO_2_ NPs composite showed the highest photocatalytic and antibacterial activity [44] (Figure 2). 

In 2019, fabrics were coated with commercial ZnO NPs, ZrO_2_ NPs, antibiotics namely doxymycin, cefadroxil and ciprofloxacin, individually and combined, in the presence of citric acid and sodium hypophosphite as a crosslinking agent and a catalyst, respectively. The results obtained signified that the antibacterial activity improved significantly and followed the decreasing order: antibiotic/MO NPs > antibiotic > MO NPs Among the used composites, doxymycin/ZnO NPs showed the highest antibacterial efficiency [45].

#### 2.1.4. Mixtures of Metal Oxide Nanoparticles

Among the metal oxides, titanium dioxide has become the benchmark for promising applications such as antibacterial agent [43]. The coating dispersion treated on textile fibers based on TiO_2_ NPs and ZnO NPs were prepared through uniform dispersion of nanoparticles in binder MTP acrylate solution by ultrasonication. It was observed the superiority of TiO_2_ NPs as antibacterial agent over ZnO NPs (against *S. aureus* bacteria). Interestingly, when equal mass ratio of TiO_2_ NPs and ZnO NPs was used, the antagonistic effect was observed in the antibacterial effect as the inhibition zone was recorded [46].

#### 2.1.5. Mixtures of Metal and Metal Oxide Nanoparticles

**Scheme 5:**
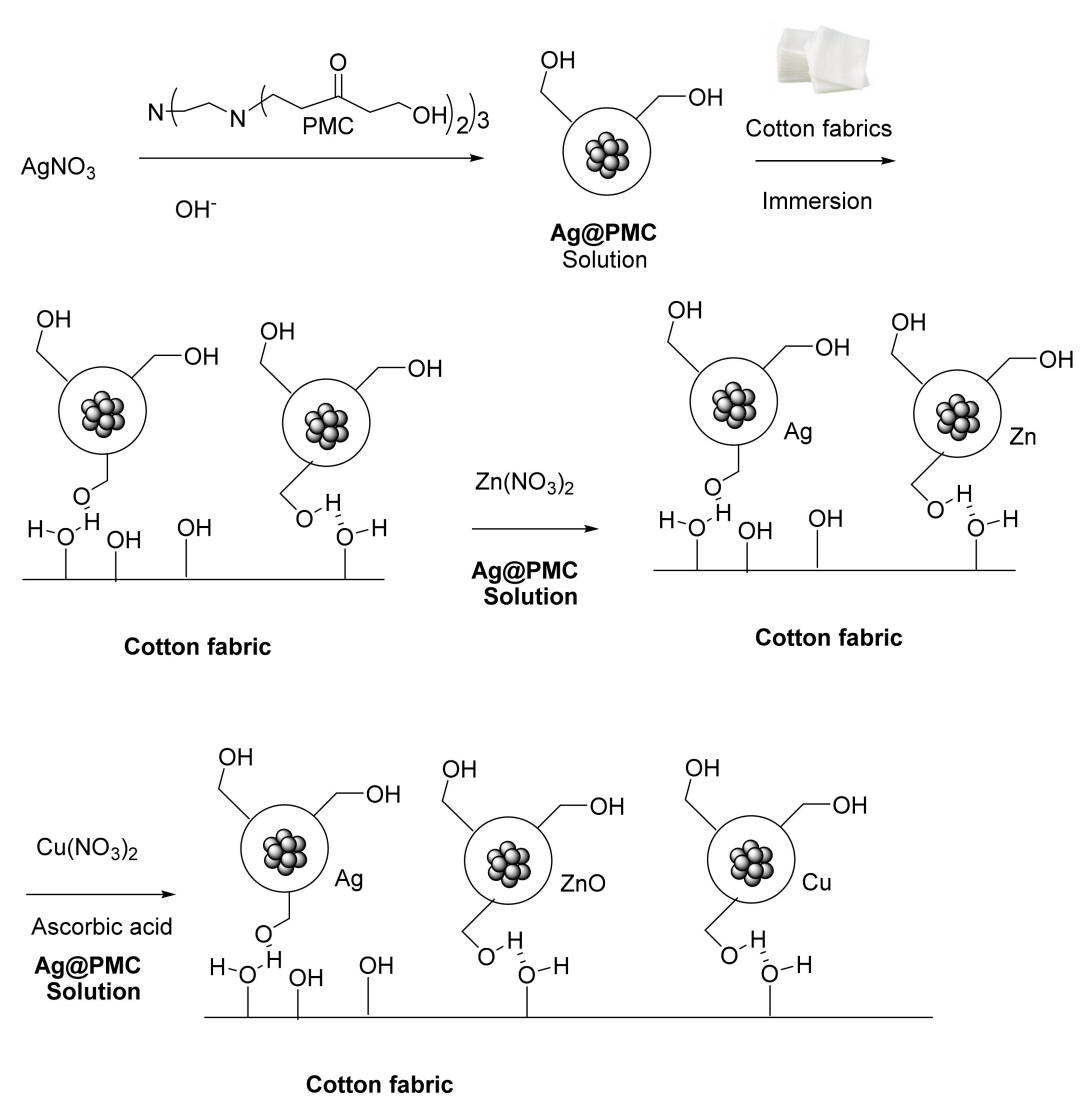
Preparation of Ag/ZnO/Cu coated cotton fabrics using a polymethylol (PMC) as reductant and stabilizing agent.

Inorganic bacterial agents are usually photocatalytic metal oxides that generally belong to semiconductor compounds. Among them, ZnO attract the most attention because its excellent biocompatibility. However, the photocatalytic antibacterial performance of ZnO can be reduced because of the high recombination rate of electron-hole pair. Moreover, the excitation light of ZnO is in ultraviolet region, which means the low utilization of daylight. One of the ways to overcome these problems and promote the photocatalytic antibacterial activity of ZnO, is doping with noble metal. On the other hand, Ag NPs are a gold standard bacteriostatic agent. Thus, El-Naggar and collaborators enhance the antibacterial activity of ZnO due to synergic charge transfer. Tri-component nanoparticles, silver, copper and zinc oxide, have been deposited onto the cotton textile surface imparting durable antibacterial activity, UV protection and conductivity properties. The chemical synthesis of these metal and metal oxide nanoparticles has been performed using a polymethylol and functionalized polyethylenimine compounds (Scheme 5) [47].

Quaternary ammonium salts are widely used in cationic polymers-based antibacterial textiles; thus, they can kill bacteria by contact mechanism [3]. In a 2020 report from Gao, Ag/ZnO nanoparticles were prepared from commercial ZnO NPs in the presence of 3-aminopropyltriethoxysilane, AgNO_3_ and trisodium citrate solution as reductant and stabilizer. Then an organic-inorganic hybrid was synthetized by radical polymerization of diallyldimethyl ammonium chloride and an acyloxy silane as coupling agent to form a pyrrolidinium based polymer. Ag/ZnO nanoparticles were incorporated to this mixture. The cotton samples were immersed in the dispersion solution for paddling. Antibacterial activity (*S. aureus* and *E. coli*), durability, hydrophilicity and air permeability were studied [48].

Recently cotton fabrics were coated with Ag, TiO_2_ and ZnO nanoparticles. Ag NPs were synthesized from AgNO_3_ with trisodium citrate, whereas TiO_2_ nanoparticles were prepared mixing TiCl_4_ and ammonium carbonate. Finally, ZnO NPs were obtained from ZnCl_2_ and sodium hydroxide. In order to decorate the cotton fabrics with the synthesized nanoparticles, the cotton fabrics were first immersed in the beaker containing a polyurethane solution. After that, the fabrics were immediately immersed in the solution containing ZnO nanoparticles and then in the beaker containing TiO_2_ nanoparticles. This process was repeated with the beaker containing Ag NPs solution. The optimum photocatalytic and antibacterial activities (*Salmonella typhi* and *Shigella*) were observed in the decorated fabrics with Ag, ZnO and TiO_2_ [49].

Another approach to obtain antibacterial cotton fabrics was the anchoring of metallic silver nanoparticles decorated with bismuth oxybromide (BiOBr) nanosheets on carboxymethyl cotton fabric. To facilitate the nucleation of BiOBr, cotton fabric was modified through S_N_2 reaction of hydroxylic groups with chloroacetic acid using NaOH as base to yield the sodium carboxylates (caboxymethyl cotton fabrics, CCF). When this fabric was immersed in a Bi(NO_3_)_3_ solution, Bi_2_O_2_^+^ ions were absorbed by the carboxyl groups. Immersion in a KBr solution induced bromide ions to react with Bi_2_O_2_^+^ forming BiOBr-CCF. Silver ions from AgNO_3_ were absorbed by BiOBr-CCF and reduced via exposure to ultraviolet irradiation. This Ag/BiOBr-CCF can effectively photodegradate rhodamine B and herbicide isoproturon in response to irradiation when placing them into solutions of these organic pollutants. Furthermore, they showed bacteriostatic effects against *E coli* and *S. aureus* [50].

Ibrahim and coworkers reported the use of *Streptomices* sp, a marine bacterial, as reducing and stabilizing agent for the biosynthesis of Au NPs. After modification of cotton fabrics with O_2_-plasma the fabrics were coated with Au NPs/ZnO NPs combination. This combination gave high antibacterial activity (*S. aureus* and *E. coli*) and durability (textiles were active after 15 washings) [51].

### 2.2. Antifungal Cotton Fabrics Containing Nanostructures

As has been previously mentioned in the introduction, it is very well known that cotton fabrics are aimed objects for fungi proliferation [4] mainly due to their great ability of retaining moisture and their large specific surface area [52]. In the past five years, much effort has been done in the preparation of functional materials for avoiding the global problem of fungi proliferation in cotton surfaces. However, literature reports of nanomaterials for cotton fabrics describing exclusively antifungal properties are scarce compared to existing ones related to biocidal properties. 

#### 2.2.1. Metal Nanoparticles

Silver is the most explored metal on cotton fabrics. Silver nitrate was impregnated through the wetness technique on siliceous matrixes dopped with carbon from spent batteries, prepared by one-pot sol-gel synthesis [53]. The hybrid NPs were applied on cotton fabric using the pad-dry-cure method. From all the prepared hybrid materials, the one with more amount of carbon in its structure showed better antifungal activity, due to an increase in the surface area with a good pore size, which expedite an effective distribution of the active phase to act inhibiting the fungi growth. The antifungal assay results were successful for *Cladisporium* sp. A direct dependence of the inhibitory effect with the amount of silver was evidenced in the tests. 

In addition, Elgorban and collaborators [54] described the synthesis of Ag NPs using the corn grain contaminant (*Nigrospora oryzae*). The antifungal activity of these biosynthesized NPs was evaluated against 8 species of *Fusaria* causing damping-off of cotton seedlings, and its antifungal activity was successfully proved.

#### 2.2.2. Metal Oxide Nanoparticles

In 2016, Djamaan and collaborators dispersed TiO_2_-SiO_2_/Chitosan NPs on cotton fabrics surface [55]. The hybrid NPs were prepared using CTAB as template of particle distributor. Different nanoparticle batches were prepared using different molar ratios of TiO_2_:SiO_2_. The prepared NPs were dispersed on cotton fabrics using the dip-spin coating technique. The antifungal activity against *A. niger* was evaluated for these fabrics. In these studies, it was clear that the amount of TiO_2_ present in these nanomaterials was crucial to achieve high activity. The hybrid TiO_2_-SiO_2_/chitosan (2:1) NPs presented the higher ability to generate hydroxyl radicals and the superoxide anion, which can destroy the fungi’s cell membranes.

### 2.3. Antibacterial and Antifungal Cotton Fabrics Containing Nanostructures

#### 2.3.1. Metal Nanoparticles

There are a good number of reports related to the antimicrobial properties of textiles. In 2016, the group of M. D. Balakumaran described the preparation of antimicrobial textiles (antibacterial and antifungal) using what they call a green chemical technology. Highly stable Ag NPs were prepared from silver nitrate using *Aspergillus terreus* Bios PTK 6 [56]. The fabrics were immersed in a solution of these Ag NPs and agitated using a rotatory shaker incubator. Then theywere squeezed using a laboratory padder at constant pressure, allowed to dry and cured. The treated cotton fabrics showed excellent antibacterial activity against. *S. aureus*, *B. subtilis*, *E. coli*, *P. aeruginosa*, *K. pneumoniae*, *MRSA* (*methicillin-resistant staphylococcus aureus*) even after 15 washings (Table 1, run 11). On the other hand, Ag NPs treated fabrics exhibited good antifungal activity against *Penicillium* sp. *A. niger* and *R. oryzae*.

In 2020, Foudas’s group described the synthesis of Ag NPs from AgNO_3_ and an endophytic actinomycetes strain of *Streptomyces laurentii* R-1 previously isolated from the roots of the plant *Achillea fragrantissima*. The AgNO_3_ solution was added to a cell-free extract and incubated. The reduction of the Ag(I) was due to the production of proteins and enzymes by the actinobacteria to reduce NO_3_^-^ to NO_2_ [57]. The Ag NPS were spherical and of varying sizes ranging 7 to 15 nm (Table 1, run 12). The cotton fabrics were padded with colloidal silver solution and showed wide spectrum activity against *S. aureus*, *B. subtilis*, *P. aeruginosa*, *E. coli* and *S. typhimurium*.

#### 2.3.2. Mixtures of Metal Nanoparticles

Eremenko and co-workers [58] developed a method for the preparation of antibacterial bandage by impregnation of cotton fabrics with aqueous solutions of silver and copper salts followed by heat treatment. The characterization showed the formation of crystalline silver NPs and bimetallic Ag/Cu composites. The bioactivity of the modified cotton was tested, showing high antibacterial and antimycotic properties with low concentrations of silver and Ag/Cu NPs. The fabrics with only Ag NPs showed antibacterial activity against a wide range of multidrug-resistant bacteria, while the fabrics coated with bimetallic NPs exhibited a pronounced antimycotic activity. The cotton fabrics with antibacterial and antifungal properties were prepared by impregnation during the washing and ironing process with Ag/Cu NPs solutions (Table 1, run 13). In 2016, Golabiewska reported the preparation and characterization of five kind of bimetallic nanoparticles, containing Ag/Cu in the forms of alloy and core-shell as well as ionic species [59]. The cotton textiles were successfully impregnated during the washing and ironing process by the as-prepared solutions to have antibacterial and antifungal properties against to *E. coli*, *S. aureus* and *Candida albicans* (Table 1, run 14). The results indicated that all tested samples after 5, 10, 15 and 20 washing/impregnated cycles exhibited an antimicrobial activity. The antifungal tests showed that only the textile impregnated with solutions containing Ag NPs/Cu^2+^ exhibited a strong inhibition of fungi growth after 15 washing/impregnation cycles. Ag NPs were prepared from Ag(OAc)_2_ with NaBH_4_ as reductant and polyvinilpyrrolidone as stabilizer and then mixed with a Cu(OAc)_2_ solution to form Ag NPs/Cu^2+^.

**Table 1:** Summary of Ag NPs-coated textiles and its bacterial effect (2016–).

Run	Synthesis Method	Source of Silver; Reductant Reagent	Additives	NP Size	Antibacterial Properties against Microorganisms	Other Properties	Ref. Author Year
1	Pad-dry-cure	AgNO_3_ *Tragacanth gum*	-	17 77 (on cotto)	*E. coli*; *S. aureus*	Water absorption	[20] Montazer 2016
2	Pad-dry-cure	AgNO_3_ Dextran	SiO_2_ VTEOS or APTEOS	10–25	*E. coli*; *S. aureus*	Thermal stability	[21] Mohamed 2017
3	Pad-dry-cure	AgNO_3_ Carboxymethyl cellulose	GPTMS DMDHEU Fluorochemical (Asahi Guard AG-925)	-	*E. coli*; *S. aureus*	Water/oil repellent Thermal stability	[22] Ibrahim 2020
4	Immersion (Dip-Dry)	AgNO_3_ Radiochemical reduction		2–10	*S. aureus*; *K. pneumoniae; MRSA*; *E. coli*; *P. aeruginosa*; *S. entérica*; *V. parahaemolyticus*	-	[23] Seino 2016
5	Immersion (Dip-Dry)	AgNO_3_ *Bacillus *sp.	N_2_-plasma treated fabric	10–17	*E. coli; S. aureus*	-	[24] Ibrahim 2017
6	Immersion	AgNO_3_ Aromatic amine (in situ polymerization)		170 and 3.5	*E. coli*; *S. aureus*	Electrical conductivity Colorimetricsensory effects	[25] Ahmed 2020
7	Immersion	AgNO_3_ NaBH_4_	EugenolSH TAMSH FQPEG	2–11	*E. coli*; *S. aureus*	-	[26] Vallribera 2019
8	Immersion (Dip-Dry)	AgNO_3_ Black rice	Carboxymethyl chitosan modified cotton	-	*E. coli;* *S. aureus*	Hydrophobicity UV protective performance	[27] He 2021
9	Ultrasonication	AgNO_3_ Cellulose mechanoradicals	-	3–40	*E. coli*.; *B. subtilis*	-	[28] Baytekin 2020
10	Aerosol-Based Process	Ag electrode Electrical discharges	-	10–150	*S. aureus*; *K. pneumoniae*	-	[29] Kruis 2016
11	Pad-Dry-Cure	AgNO_3_ *Aspergillus terreus*	-	8–20	*S. aureus*; *B. subtilis;* *E. coli*; *P. aeruginosa*; *K. pneumoniae*; *MRSA*	Antifungal	[56] Balakumaran 2016
12	Pad-Dry-Cure	AgNO_3_ endophytic actinomycetes strain of *Streptomyces laurentii*	-	7–15	*S. aureus*; *B. subtilis*; *P. aeruginosa*; *E. coli*	Anticancer Antifungal	[57] Fouda 2020
13	Immersion	AgNO_3_ Ironed at 220 °C	-		*E. coli*, *E. aerogenes; P. mirabilis*; *K. pneumoniae*	Antifungal	[58] Eremenko 2016
14	Impregnation by Pressing at 200 °C	Ag(OAc)_2_ NaBH_4_	Polyvinyl pyrrolidone	18	*E. coli*; *S. aureus*	Antifungal	[59] Golabiewska 2016

#### 2.3.3. Metal Oxide Nanoparticles

**Scheme 6:**
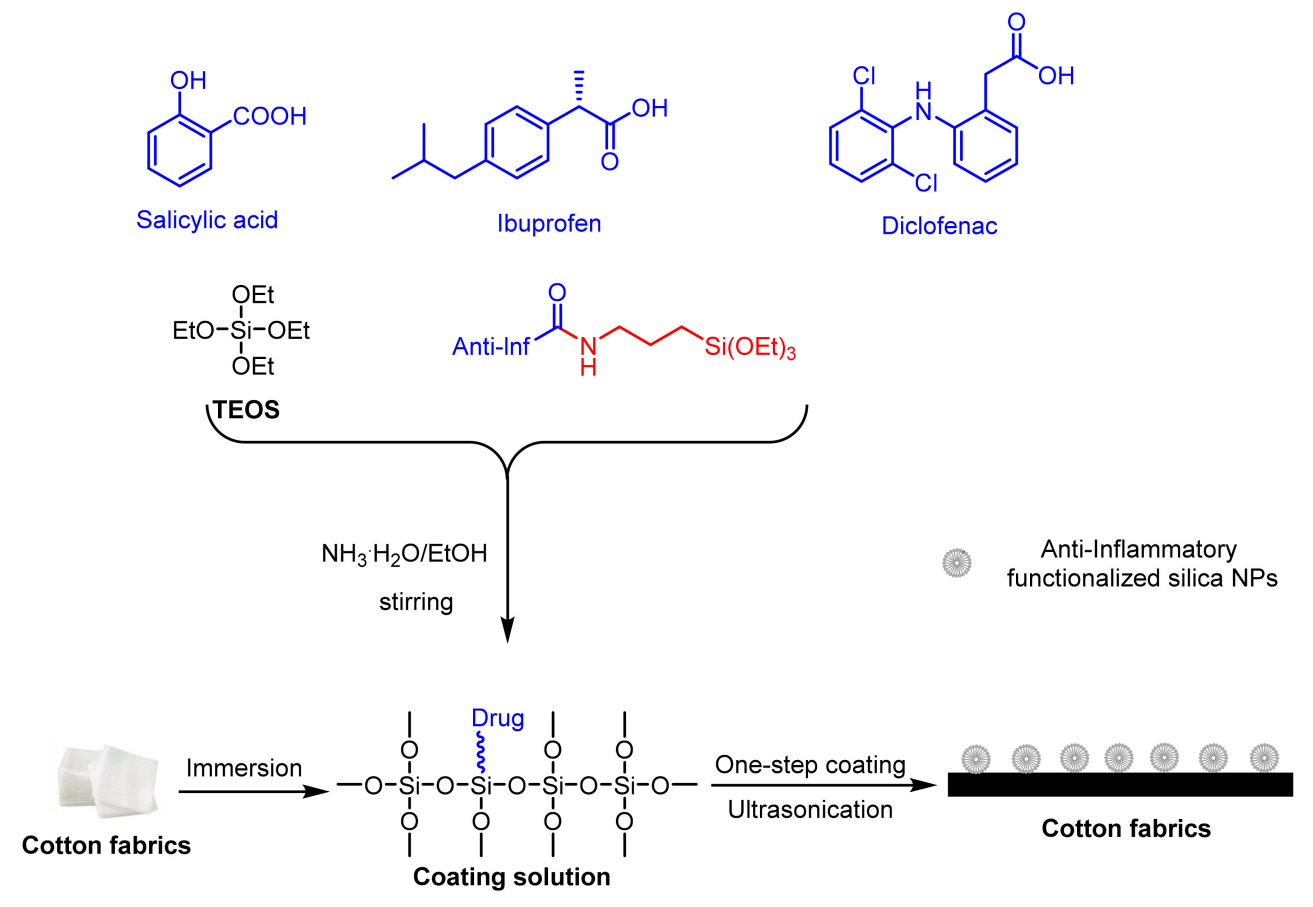
Silica sol preparation and coating procedure for highly hydrophobic cotton fabrics.

Iron oxide NPs were prepared though sonochemistry [60], thanks to the generation of oxidative species during the acoustic cavitation in water and to the formation of hydrogen peroxide. On this basis, the process began with Fe^2+^ salt as iron source, part of which was oxidized to Fe^3+^ via radicals formed though the water sonolysis under ultrasound irradiation instead using two iron precursors (Scheme 6). Then, due to the presence of cotton fabric and its sonication, the surface cellulosic fabric provides sites for Fe_3_O_4_ NPs to be nucleated and grown during the processing time at 80 °C under sonication (Table 2). The authors claim that the alkaline conditions of the process not only serve as important factor for the Fe NPs, but it plays another prominent role in surface activation of cotton, forming hydroxyl and carboxyl groups, which could be the possible chemical bonding between the fabrics and the nanomaterials. The modified cotton fabrics were tested successfully against *S. aureus* (antibacterial test) and *C. albicans* (antifungal test), showing an efficacy of 95% and 99%, respectively.

Ibrahim et al. reported the effect of different stabilizing agents on the preparation of ZnO NPs from Zn(OAc)_2_ and then calcination. They investigated the influence of soluble starch, lactose, carboxymethyl cellulose, urea and polyvinylpyrrolidone. They showed excellent antibacterial (*E. coli* and *S. aureus*) and antifungal properties (*A. niger*) [61].

In 2017, El-Nahhal and co-workers [62] prepared and deposited ZnO nanoparticles on cotton fabrics via ultrasound irradiation. The mechanism of formation of the ZnO-NPs by addingZnSO_4_·7H_2_O into the solution containing a surfactant and sodium hydroxide probably involves three or four steps, the first one being the interaction between Zn^2+^ and the surfactant and the second step the formation of stable Zn(OH)_2_ which is absorbed onto the cotton surface. Thirdly, the transformation of Zn(OH)_2_ to ZnO-NPs occurs by ultrasound irradiation. Finally, the removal of the excess of reactants, including the surfactant, is accomplished by washing process The ZnO NPs (from 8 to 12.8 nm) were successfully stabilized by using different surfactants (sodium dodecyl sulfate (SDS), cetyl trimethyl ammonium bromide (CTAB), triton X-100 (TX-100) and alkyl hydroxyethyl dimethyl ammonium chloride (HY)). These modified fabrics proved to be effective against *E. coli* and *S. aureus* after ten wash cycles, being the ZnO-SDS and Zn-HY the most effective. ZnO-NPs coated cotton has greater activity against the bacterial species compared with the fungal species (*C. albicans* and *M. canis*). One year later, the same research group studied the efficacy of surfactants for the deposition of copper oxide NPs onto cotton surface [63]. The use of surfactants provided better adhesion of the CuO NPs, thus enhancing the coating durability on fibers surfaces. These modified cotton fabrics resisted ten washing cycles, in the presence of SDS and TX-100 a loss of 25% and 66.5% of CuO was detected, respectively. The most effective modified cotton fabrics against *S. Aureus* and *E. Coli* strains were the ones modified by CuO/SDS NPs and CuO/HY NPs. The authors claim that this success is due to the small mean crystallite size of these nanostructures. Once more, CuO Nps coated cotton has greater antibacterial activity compared with the antifungal’s activity (*C. albicans* and *M. canis*).

In the field of ZnO NPs, Gafur and co-workers prepared ZnO NPs and coated cotton fabrics using the dip coating technique [64]. Several cotton fabrics were prepared by varying the concentration of solutions of ZnO NPs (1, 2, 2.5 and 3 M). They tested these modified cotton fabrics against *S. aureus*, *E. coli* and *A. niger* to check the antibacterial and antifungal activity. The 2 M ZnO NPs coated cotton showed the highest zone of inhibition for the three studied stains. 

In 2020, a novel impregnation method for the preparation of cotton nanocomposite with strong antimicrobial activity against bacteria and yeast was developed [65]. The process involved the treatment of the cotton fabric with a 4 wt% sol of the hydrolyzed (3-aminopropyl)triethoxysilane in ethanol by a pad-dry-cure method as a first step, and then the synthesis of CuO/Cu_2_O NPs on the fabric surface as a second step. Two batches were prepared, varying the amount of copper (1 and 10 mM). The NPs formation was based on the adsorption of copper (II) cations by the introduced amino groups through the sol-gel process, and these absorbed cations were reduced by sodium borohydride. Since this modified cotton could be exploited in the wound dressing production, several microorganisms were tested, including drugs-resistant bacteria and yeast. As expected, the antibacterial activity against *E. coli* and *S. aureus* strongly depended on the copper content on the modified cotton, achieving maximum reduction of bacteria proliferation, while the antifungal activity against *C. albicans* was worse in terms of activity. The authors tested the cytotoxicity of these fabrics because is an important parameter that verifies the potential application in wound dressing. Again, the metal content was critical, being the one with less copper content the less toxic for skin cells.

Gowri and collaborators [66] described the synthesis of TiO_2_ nanoparticles using extracts derived from Aloe vera plant. The source of titanium was titanium tetraisopropoxide, that was treated with the aqueous extract gel and then calcinated al 500 °C. The cotton fabric was immersed in the solution containing TiO_2_ nanoparticles and citric acid, a crosslinking agent. Coating was carried out by pad-dry-cure method. These biosynthesized nanoparticles presented different biological applications. Upon dispersing these NPs on cotton fabrics, the antifungal activity was tested. These TiO_2_ NPs showed inhibitory power against *C. albicans* and *A. niger* strains from agar diffusion method. The antifungal power of these nanomaterial is explained by their disruption ability of cell membrane thereby the release of protein, carbohydrate and lipids through the damaged cell membrane. In addition, the results showed that the coated fabric presented higher antibacterial against *S. aureus* than to *E. coli* probably due to the attraction of generated active oxygen species of TiO_2_ with *S. aureus*.

In 2019, functional cotton was prepared applying TiO_2_ NPs and green walnut shell dye to modify the metal oxide NPs and achieve natural dye coloration [67]. The aim of this material is to provide coloration and functional finishing of cotton fabric, such as self-cleaning properties, antifungal and antibacterial activities. In this study, the comparison in terms of antibacterial and antifungal activity between raw cotton, cotton treated with TiO_2_ NPs and cotton treated with TiO_2_ NPs in the presence of the natural dye walnut shell was developed. Both, the antibacterial (tested in *S. aureus* and *E. coli* stains) and antifungal (tested in *C. albicans*) activities were enhanced in the cases of modified TiO_2_ NPs with the natural dye. The authors attribute this synergistic effect to the presence of phenolic and naphthoquinone compounds, present in walnut shell dye’s structure.

#### 2.3.4. Mixtures of Metal Oxide Nanoparticles

Chitosan-g-polyvinyl acetate (Cs-g-PVAc) was synthesized successfully using redox copolymerization using potassium persulfate as initiator. Two samples from nanocrystalline [TiO_2_]_1−x_[ZnO]_x_ (where x = 0, 0.2) were synthesized by sol-gel method using stoichiometric amounts of titanium isopropoxide (TIPP) and zinc acetate dihydrate as starting raw materials. TiO_2_ and TiO_2_ doped with ZnO were used for preparing an emulsion from polymer–metal oxide nanocomposites. Cotton fabrics were treated with the prepared emulsions using citric acid and sodium hypophosphite. The bacteria *S. aureus*, *P. aeruginosa* and the fungi *A. niger* were selected to evaluate the antimicrobial activities. All the treated fabrics had antimicrobial activity higher than non-treated cotton fabrics as a result of the presence of chitosan in addition to the metal oxide nanoparticles [68].

#### 2.3.5. Mixtures of Metal and Metal Oxide Nanoparticles

Different combined functionalizations have been studied in order to obtain multifunctional textiles. It is known that the modification of silica sol with nanoparticles of aluminum trioxide imparts resistance to abrasion to fabrics. The addition of TiO_2_ provides excellent UV-radiation protection especially in the region of 290–315 nm probably due to the scattering effect of UV radiation by the TiO_2_ particles. Furthermore, TiO_2_ photocatalytic properties imparts self-cleaning capability to the cotton fabric. In addition, the presence of phosphorus compounds yield flame retardancy properties.

Multifunctional thin-coating textile finishing with the use of hybrid Al_2_O_3_/SiO_2_ modified with Ag/Cu NPs and TiO_2_ P25 were prepared in 2018 [69]. An elastic xerogel coating on the fabric surface was achieved depositing the modified hybrid Al_2_O_3_/SiO_2_ by the padding method, followed by drying and thermal heating. The hybrid Al_2_O_3_/SiO_2_ sol was previously prepared using as precursors 3-glycidoxypropyl)trimethoxysilane and aluminium(III) isopropoxide. Then, commercial alloy Ag/Cu nanoparticles and TiO_2,_ were dispersed in the hybrid Al_2_O_3_/SiO_2_ sol. This modified sol was applied on the fabric surface by the padding method. The addition of Cu, TiO_2_ and Ag allowed the authors to obtain functionalized cotton with microbicidal effect. The experiments showed good microbiological activity with the xerogel coated with these nanoparticles against bacteria (*S. aureus* and *E. coli*) and fungi (*Candida albicans* and *A. niger*). The addition of TiO_2_ has no negative effect on the bioactive properties compared with the hybrid material with only Cu and Ag. So, at the same time, the use of these two metal nanoparticles can contribute to create a synergistic effect whereby the modified cotton will show bioactive properties in the visible light (due to Cu and Ag) as well as under the influence of UV light (due to the addition of TiO_2_ P25).

Gao’s research group prepared silver/zinc oxide (Ag/ZnO) nanoparticles with different molar ratios of silver by chemical precipitation method [70]. First, the ZnO and Ag NPs were independently prepared. The morphology of the ZnO NPs were rod-like (170 nm length and 30 nm in diameter) while the Ag NPs were spherical (15 nm diameter). The addition of silver in the preparation of the ZnO NPs did not change the structure. The purpose of these hybrid nanostructures was to obtain treated cotton fabrics with excellent performance in terms of hydrophobicity, UV resistance, antibacterial and anti-mildew activity. The antimicrobial activity of the cotton fabrics was evaluated against bacteria (*S. aureus*, *E. coli*) and fungi (*C. albicans*). The results showed a silver content dependence, as much silver content the better antibacterial and antifungal activity. The antifungal activity of these fabrics was also evaluated against *A. flavous*. In order to have anti-mildew properties, the amount of silver in these NPs must be at least 1%, being 3% the best one to achieve a proof grade 1 (a proof grade of 4 means no mildew resistance). So, Ag/ZnO (3% Ag) NPs on cotton fabrics have an effective antibacterial activity and gave good mildew resistance.

#### 2.3.6. Miscellaneous Nanoparticles

An effective ultrasound assisted deposition of vanillin NPs, raspberry ketone NPs and camphor NPs on textiles was reported by Gedanken’s group [71]. These nanomaterials were coated on cotton fabrics and showed excellent antibacterial and antifungal activity on cotton bandages and polypropylene surfaces. They exhibited excellent bioactivity against *E. coli*, *S. parathyphi A* and *C. albicans*. The authors highlight the edibility of these fragrant materials, making then useful for packaging.

### 2.4. Anti-Inflammatory Cotton Fabrics Containing Nanostructures

The synthetic anti-inflammatory corticosteroid betamethasone sodium phosphate (BSP) has been loaded via postimpregnation on mesoporous silica nanoparticles of SBA-15 type, previously modified with (3-aminopropyl)triethoxysilane (APTS). These drug-loaded silica particles (SBA-15-NH_2_-BSP) have been grafted on the cotton fabric surface using the bacteriostatic polysaccharide chitosan and polysiloxane reactive softener as a soft and safe fixing, with the aim of developing a cotton fabric with anti-inflammatory drug delivery properties. Scanning electron microscopy (SEM) images confirmed the presence of silica particles on the surface of cotton fibres. A decrease of tensile strength and air permeability were also indicative of the presence of silica/chitosan/softener coating on the fabric surface. The release profile of BSP in phosphate buffer saline (PBS) solution at pH 7.2–7.4 was analyzed using UV-Vis spectra. The application of chitosan and softener lead to a lower release rate of BSP from mesoporous silica particles. Additionally, the cotton textile exhibited a powerful antibacterial activity due to chitosan. Cytotoxic assays revealed the nontoxicity of the silica particles against the blood cell [72].

Recently, we have performed the coating of cotton fabrics with dense silica nanoparticles covalently functionalized with carboxyl-containing nonsteroidal anti-inflammatory drugs (salicyclic acid, ibuprofen, diclofenac) through amide bonds [17]. One step-coating method was used as depicted in Scheme 6. The coating solutions were obtained by co-condensation of TEOS with the corresponding organosilane in aqueous ammonia in ethanol under stirring at room temperature for 12 h. After ultrasonication of the milky solution for half an hour to produce a homogeneous suspension, a piece of cotton fabrics was immersed in this suspension and ultrasonicated for another 30 min. The cotton textile was removed, washed and dried. In this way, the roughness of the surface was increased, providing hydrophobicity to the modified fabrics (contact angle 140.5 °). The presence of drug functionalized nanoparticles on the surface of the cotton was ensured by scanning electron microscopy (SEM), X-ray photoelectron spectroscopy (XPS), energy-dispersive x-ray spectroscopy (EDX) and element mapping. SEM and EDX were taken on a SEM Zeiss Merlin with and INCA detector from Oxford Instruments (England) and XPS with a SPECS PHOIBOS 150 hemispherical analyzer (SPECS GmbH, Berlin, Germany).

**Scheme 7:**
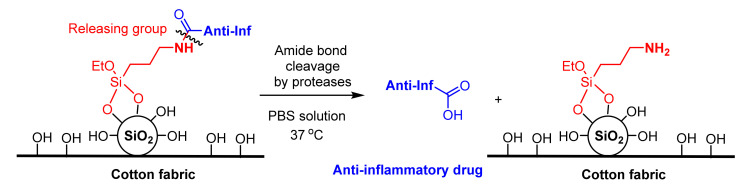
Release of anti-inflammatory drug by enzymatic cleavage of amide bond.

The treatment of these coated cotton fabrics with model proteases and leucocytes from rat blood resulted in the in situ release of the anti-inflammatory agent by selective enzymatic cleavage of the amide bond (Scheme 7).

**Scheme 8:**
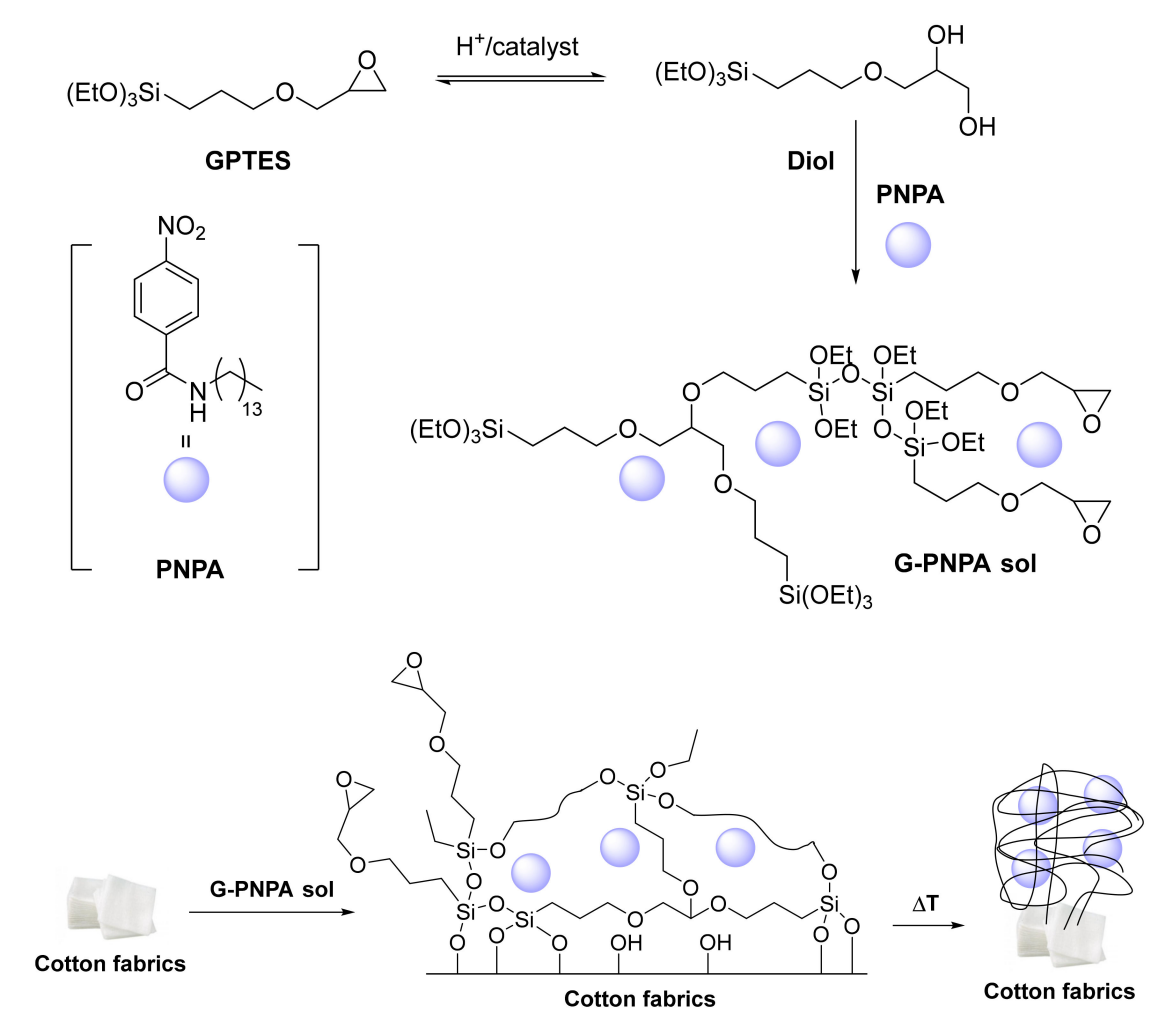
Formation of G-PNPA sol and its application on cotton surface.

A nano-hybrid sol-gel coating has been developed and used as an entrapping polymeric cross-linked network for molecules with anti-inflammatory and antioxidant properties such as PNPA. The obtained G-PNPA functional sol has been applied by padding on cotton fabrics and thermally cured (Scheme 8). Under the action of a cutaneous stimuli, a controlled release of the drug has been achieved [73].

Diphenylamine (FF) peptide nanotubes loaded with the antimicrobial and anti-inflammatory agent curcumin have been deposited on cotton fabrics by a sonochemical method. Good adherence of the nanotubes to the fabrics is due to physical adsorption because of the sonication process. The kinetics profile of the curcumin release has been monitored using absorbance analysis at 430 nm. The sonochemical method has been found important for the durability of the coating upon washing. Moreover, both nanotube adherence to the fibers and curcumin release were modulated by the sonication time [74].

## 3. Conclusions

The surface modification of cotton fabrics with nanostructures to provide antibacterial properties to the textiles has been extensively studied in the last five years. Thus, metal nanoparticles (mainly Ag NPs but also, in a lesser extent, Au NPs and mixtures of Ag/Cu or Ag/Au/Pt) have been used for that purpose, together with metal oxide nanoparticles (mainly ZnO NPs, but also CuO, TiO_2_, SiO_2_, Fe_3_O_4_, mixtures of oxides such as TiO_2_/ZnO, CuO/Cu_2_O) or mixtures of metal and metal oxide nanoparticles (such as Ag/ZnO, Ag/Cu/ZnO, Ag/TiO2/ZnO). On the other hand, only few reports are available with respect to the coating of cotton fabrics with nanostructures (Ag NPs, TiO_2_/SiO_2_/chitosan) aimed exclusively to avoid fungi proliferation. In some cases, both antibacterial and antifungal activities have been reported. The antibacterial activity of the functionalized cotton has been mainly determined against *E. coli* (gram negative) and *S. aureus* (gram positive) and the anti-fungal activity against *Candida albicans* and *A. niger*.

For the synthesis of these nanoparticles, the common reducing agents (such as NaBH_4_) used in the traditional chemical reduction of metal salts, are being largely replaced by more eco-friendly and safe reductants such as biopolymers or compounds derived from marine bacteria, fungi, algae, extracts from red sand and from different plants. Formation of ZnO and Fe_3_O_4_ NPs by ultrasound irradiation has also been described. The coating of cotton is mostly performed by pad-dry-cure, dip-spin coating or by immersion (dip-dry) methods, the last one sometimes with the aid of ultrasonication. The use of polymeric binders (such as PHDMS or MTP), cross-linking agents (such as DMDHEU, BTCA or citric acid), composites with derivatized silica nanoparticles or siliceous matrixes, several surfactants as well as ultrasound techniques results in better adhesion of the nanostructured coating and considerable enhancement of the durability of the treated textile. 

On the contrary, although very promising for the treatment of chronic wounds, the topic of anti-inflammatory functionalized cotton fabrics has been much less explored, and a scarce number of reports have been found from 2016 concerning nanostructured coatings. Thus, anti-inflammatory agents (betamethasone, curcumin, PNPA) have been loaded on modified mesoporous silica nanoparticles, on diphenylamine peptide nanotubes or in a nano-hybrid sol-gel coating and grafted or deposited on cotton fabric surface with the aim of drug-releasing. A different approach has been recently described concerning the coating of cotton textile with dense silica nanoparticles covalently functionalized with carboxyl-containing nonsteroidal anti-inflammatory drugs through amide bonds. In-situ release of the drug takes place by selective enzymatic cleavage of the amide bond by proteases. 

Following with the tendency of the last years, an increase of the application of nanostructured coatings for the obtention of multifunctional textiles with improved properties is expected in the near future.

## Figures and Tables

**Table 2:** Formation of iron oxide nanoparticles through sonochemistry.

${\mathrm{FeSO}}_{4}\to {\;\mathrm{Fe}}^{2+}+{\;\mathrm{SO}}_{4}^{2-}$
$H_{2}O\;\overset{\mathrm{Sonication}}{\to }\;H{•}^{}+•\mathrm{OH}$
$H{•}^{\;}+•H\;\to {\;H}_{2}$
$\mathrm{OH}{•}^{}+\;\mathrm{OH}{•}^{}\to {\;H}_{2}O_{2}$
$2{\;\mathrm{Fe}}^{2+}+{\;H}_{2}O_{2}\to \;2{\;\mathrm{Fe}}^{3+}+2{\;\mathrm{OH}}^{-}$
${\mathrm{Fe}}^{2+}+{\;\mathrm{OH}}^{-}\to \;\mathrm{Fe}{\left(\mathrm{OH}\right)}_{2}$
${\mathrm{Fe}}^{3+}+{\;\mathrm{OH}}^{-}\to \;\mathrm{Fe}{\left(\mathrm{OH}\right)}_{3}\to \mathrm{FeOOH}$
$\mathrm{Fe}{\left(\mathrm{OH}\right)}_{2}+2\;\mathrm{FeOOH}\;\to {\;\mathrm{Fe}}_{3}O_{4}+2{\;H}_{2}O$
